# Physiological Ventricular Pacing from the Right Side of the Septum by Engaging the Subendocardial Purkinje Network

**DOI:** 10.19102/icrm.2025.16025

**Published:** 2025-02-15

**Authors:** Atul Prakash, Iyad Farouji, Richard Sutton

**Affiliations:** 1 Department of Cardiology, St Mary’s General Hospital, Newark, NJ, USA; 2 Department of Cardiology, St Michael’s Medical Center, Newark, NJ, USA; 3 Department of Cardiology, Hammersmith Hospital Campus, National Heart & Lung Institute, Imperial College, London, UK

**Keywords:** Characteristic ventricular potential, narrow paced QRS, novel method to achieve physiological pacing, physiological pacing, right ventricular septal pacing

## Abstract

Right ventricular (RV) pacing, particularly from the RV apex, causes bilateral ventricular dyssynchrony, reducing systolic and diastolic function, by delayed activation of the lateral left ventricle, resulting in a wide QRS with a left bundle branch block (LBBB) morphology. Alternative pacing strategies, such as His-bundle pacing and LBB area pacing, tend to be more physiological, avoiding this problem. The feasibility of attaining a narrow paced QRS from the RV septum has not been methodically examined. This study aimed to test the hypothesis that, through pacing at select RV septal sites by careful mapping, it is possible to achieve a narrow “paced QRS,” facilitating physiological pacing. The underlying assumption is that a narrow paced QRS prevents long-term deterioration of cardiac function. During dual-chamber pacemaker implantation with standard active fixation leads, the RV septum was mapped carefully before fixing the lead. A characteristic spike potential was identified at some sites which, on stimulation, yielded a narrow paced QRS. The paced QRS duration was measured at different mapping sites; the narrowest paced complex was chosen for long-term pacing. Sixteen consecutive patients underwent pacemaker implantation using this mapping technique. A narrow paced QRS was achieved in 12 patients, whereas narrow paced complexes could not be achieved in 4 patients. Among the 12 narrow paced QRS patients (mean age, 81.5 ± 8.2 years), the indication for pacing was atrioventricular block in 6 patients and sick sinus syndrome in 6 patients. Two patients showed a negative paced QRS in leads 1 and aVL, suggesting an early left-sided septal activation. In the 12 narrow paced QRS patients, the post-pacing mean QRS duration (121.5 ± 14.9 ms) was not significantly different from the pre-pacing mean QRS duration (118.2 ± 23.5 ms) (*P* > .5); the QRS morphology was normal in seven patients, while four patients had LBBB and one patient had right bundle branch block. In all 12 patients, the narrowest paced complex was associated with a characteristic potential in the endocardial electrogram. Detailed RV septal mapping can yield a narrow paced QRS associated with a characteristic endocardial potential in the pre-pacing electrogram, suggesting possible direct native conduction system access.

## Introduction

It is well established that right ventricular (RV) pacing, especially at its apex, creates dyssynchrony with deterioration of systolic function,^1^ which stems from the delayed activation of the left ventricle—importantly, the lateral basal wall—resulting in a wide QRS. The deterioration of left ventricular (LV) systolic function and the possibility of diastolic function long term may be the result of prolonged total activation time in the ventricle as well as a change in the activation sequence or both.^2^ In an attempt to improve and prevent deterioration of LV systolic function, the addition of an LV lead positioned via the coronary sinus was first considered to resynchronize the ventricles.^3^ Subsequently, His-bundle pacing was introduced to minimize the long-term detrimental effects of RV pacing.^4^ This, however, was complicated by dislodgement and relatively high stimulation thresholds.^5^ Left bundle branch area (LBBA) pacing is now recommended to achieve a similar result.^6^ His-bundle pacing and LBBA pacing require special leads and delivery sheaths to achieve satisfactory pacing.^7^ These sites of stimulation also demand longer procedure times.^8^ Based on septal anatomy and the distribution of the conduction system, we evaluated the possibility of alternative RV septal sites, which would achieve a similar result without requiring lead delivery systems. This, of course, assumes that a narrow QRS implies engaging the intrinsic conduction system and that a change in morphology within a narrow QRS may have less importance than an abnormal ventricular activation sequence.

### Objectives

The primary objective of the study was to determine the feasibility of attaining a narrow paced QRS from the RV septum to facilitate physiological pacing. The secondary objective was to identify the characteristics of the recorded electrogram, especially the current of injury at an optimal site.

## Methods

### Right ventricular lead placement

Access was obtained via cephalic vein cutdown or percutaneous puncture of the axillary or subclavian vein. A 52-/56-cm standard active fixation lead was passed into the RV. Using a curved stylet (by hand from a straight stylet), different sites on the RV septum, apex, inflow and septal outflow tract, and mid-septum were mapped. The positions were viewed in right anterior oblique and left anterior oblique (LAO) projections. The apical septal and low septal locations were usually reached by a straight stylet or a very slightly curved stylet. The curve was then accentuated once, and the mid-septal and outflow tract locations were reached by a variation of the stylet within the lead. Different locations could also be reached by withdrawing the stylet a few centimeters and then reintroducing with its full length once the location was reached.

**Figure 1:**
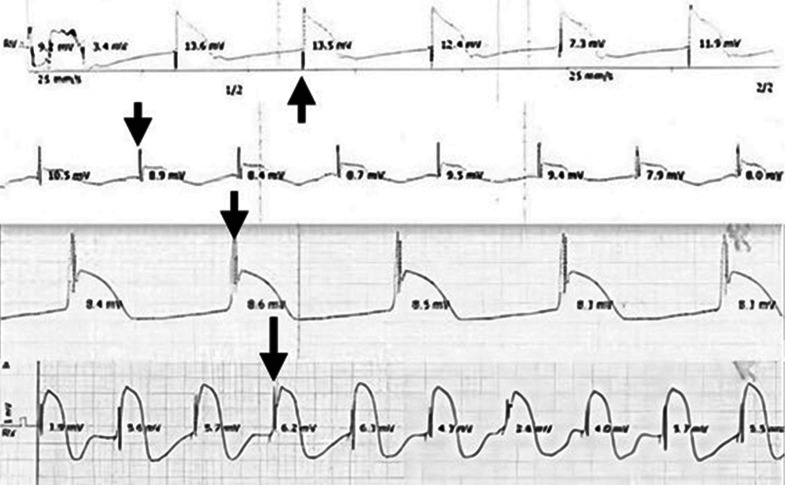
Bipolar endocardial electrograms obtained from the ventricular pacing lead from the interventricular septum during mapping high-frequency potential (sharp upstroke spike) at the onset of the current of injury (black arrows).

Mapping was initially performed using a bipolar endocardial electrocardiogram (ECG), seeking a characteristic potential. The optimal site was identified by demonstrating a high-frequency spike potential either at the onset or just after the onset of the current of injury and a narrow paced QRS complex on the surface ECG **(Figure 1)**. The potential identified was a high-frequency deflection timing either at the onset or after the current of injury. Based on the timing and the absence of any atrial recording felt to be distinct from a His-bundle or a right bundle potential, stimulation at this site resulted in the narrowest paced QRS, compared with other sites, on a 12-lead ECG. The QRS width was measured at all these locations. The standard helix was extended at the optimal site to fix the lead. The paced QRS duration was again measured to check that it had not changed. Standard sensing and pacing thresholds were measured.

During mapping and lead placement, we were particularly cautious when positioning the lead in patients with persisting left bundle branch block (LBBB), aware that complete heart block could be precipitated by even minor right bundle branch trauma. An atrial lead was also implanted in the interatrial septum using the LAO view. The stylet for this lead had a reverse curve at its end in addition to the standard curve (for an appendage approach). The lead was fixed by helix extension, and routine tests were performed. The procedure and fluoroscopy times were routinely recorded.

Written informed consent was obtained from all patients, including for collecting the implant data. The placement of the RV lead at the septum and searching for a narrow QRS has been the practice of the operator for all pacemaker implants. This series, however, had a standardized approach, with recordings documented at all sites mapped.

This was an anonymized clinical observational study during standard pacemaker implantation, implying no ethical issues.

### Statistical analysis

All calculation and hypothesis tests were performed using the R software version 3.4.1 (R Project for Statistical Computing, Vienna, Austria). A paired *t* test was performed to test the null hypothesis that the true mean of the differences between pre- and post-QRS durations was equal to 0 s. We calculated that, if using paired data, we would require a sample size of 11 patients in order to detect a mean difference of 5 ms with an 85% power at a significance level of .05.

## Results

Sixteen consecutive patients underwent pacemaker implantation for standard indications. Among them, this mapping technique successfully achieved a narrow paced QRS in 12 patients. **Table 1** presents patients’ demographics, characteristics, and ECG findings before and after pacing. **Figure 1** illustrates the characteristic potential in four patients. **Table 2** presents the details of the four patients in whom the sought characteristic potential could not be found and a narrow paced QRS endpoint could not be achieved. In each of these patients, the narrowest paced QRS was >30 ms wider than the intrinsic QRS. For the 12 former patients, the indication for pacing was atrioventricular block in 6 patients and sick sinus syndrome in 6 patients; their mean age was 81.5 ± 8.2 years. Comorbidities included hypertension and coronary artery disease; ECG before pacing showed a normal QRS duration in seven patients, RBBB morphology in three patients, LBBB in one patient, and intraventricular conduction delay in one patient **(Table 1)**.

**Table 1:** Patient Characteristics, Pacemaker Indications, and ECG Changes with Pacing

Number	Age (Years)	Sex	Comorbidities	Indication	QRS Before	Baseline Morphology	QRS After	Axis	Morphology After	aVL	Lead 1	aVR	Lead Location
1	80	Female	HTN, DM, DLP	Second-degree AV block, Mobitz II	90 ms	Normal	108 ms	Left	LBBB	Positive	Positive	Negative	RVOT
2	71	Male	HTN, CVA	Symptomatic 2:1 AV block	102 ms	Normal	120 ms	Left	LBBB	Positive	Positive	Negative	Mid-septum
3	72	Female	ESRD, anemia, hypertension, type 2 diabetes, GERD, hypothyroidism, COPD, CAD, Parkinson’s disease	Third-degree AV block	103 ms	Normal	101 ms	Normal	Normal	Positive	Positive	Negative	RVOT
4	86	Male	Paroxysmal atrial fibrillation, angina	Third-degree AV block	141 ms	LBBB	137 ms	Left	LBBB	Positive	Positive	Negative	RVOT
5	84	Female	Hypertension, SVT, CKD stage 3, CAD	Sinus pause	133 ms	RBBB	133 ms	Left	LBBB	Negative	Negative	Positive	RVOT
6	74	Female	Type 2 diabetes, hypertension, syncope, hyperlipidemia, hypothyroidism	Third-degree AV block	97 ms	Normal	100 ms	Left	Normal	Positive	Positive	Negative	Mid-septum
7	80	Male	CAD, DLP, AF	SSS	155 ms	RBBB, LAFB	122 ms	Left	RBBB	Positive	Positive	Negative	Mid-septum
8	82	Female	HTN, DLP	Sinus pause	72 ms	Normal	94 ms	Normal	Positive	Positive	Negative	Negative	Mid-septum
9	96	Male	HTN, DM, DLP CAD	SSS	140 ms	Normal	148 ms	Normal	Negative	Positive	Negative	Positive	Mid-septum
10	73	Male	HTN, DM, CKD	SSS	90 ms	Normal	124 ms	Normal	Normal	Positive	Positive	Negative	RVOT
11	82	Male	CAD, HTN	SSS	111 ms	Normal	120 ms	Left	Normal	Positive	Negative	Negative	Mid-septum
12	78	Female	HTN, DM, CKD, stroke	Third-degree AV Block	135 ms	RBBB	131 ms	Normal	Normal	Positive	Positive	Negative	RVOT

*Abbreviations:* AF, atrial fibrillation; AV, atrioventricular; CAD, coronary artery disease; CKD, chronic kidney disease; COPD, chronic obstructive pulmonary disease; CVA, cerebrovascular accident; DLP, dyslipidemia; DM, diabetes mellitus; ECG, electrocardiogram; ESRD, end-stage renal disease; GERD, gastroesophageal reflux disease; HTN, hypertension; LAFB, left anterior fascicular block; LBBB, left bundle branch block; RBBB, right bundle branch block; RVOT, right ventricular outflow tract; SSS, sick sinus syndrome; SVT, supraventricular tachycardia.

**Table 2:** Patient Characteristics, Pacemaker Indications, and ECG Changes with Pacing Where a Narrow QRS Was Not Achieved

Number	Age (Years)	Sex	Comorbidities	Indication	QRS Before	Baseline Morphology	QRS After	Axis	Morphology After	aVL	Lead 1	aVR	Lead Location
1	70	Male	Epilepsy, DLP, HTN	SSS	90 ms	Normal	190 ms	Left	LBBB	Positive	Positive	Negative	Low septum
2	89	Female	DLP, HTN	Second-degree AV block, Mobitz	95 ms	Normal	156 ms	Normal	IVCD	Positive	Positive	Negative	RVOT
3	85	Male	DLP, HTN, DM, CAD	Second-degree AV block, Mobitz	106 ms	Normal	164 ms	Normal	LBBB	Negative	Negative	Negative	Mid-septum
4	74	Female	DM, DLP, HTN, hypothyroidism	SSS	97 ms	Normal	142 ms	Left	LBBB	Positive	Positive	Negative	Mid-septum

*Abbreviations:* AV, atrioventricular; CAD, coronary artery disease; DLP, dyslipidemia; DM, diabetes mellitus; ECG, electrocardiogram; HTN, hypertension; IVCD, intraventricular conduction delay; LBBB, left bundle branch block; RBBB, right bundle branch block; RVOT, right ventricular outflow tract; SSS, sick sinus syndrome.

**Figure 2:**
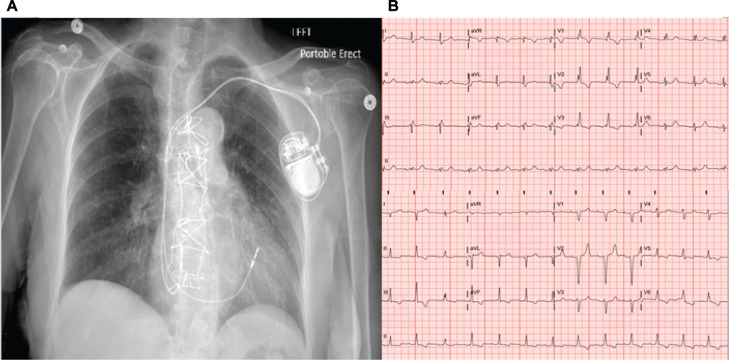
Right ventricular lead position and QRS axis shift in the first patient. **A:** Chest X-ray anteroposterior view with the final right ventricular lead location. **B:** Electrocardiograms before (top) and after (bottom) pacing, showing a QRS axis shift in leads I and aVL.

**Figure 3:**
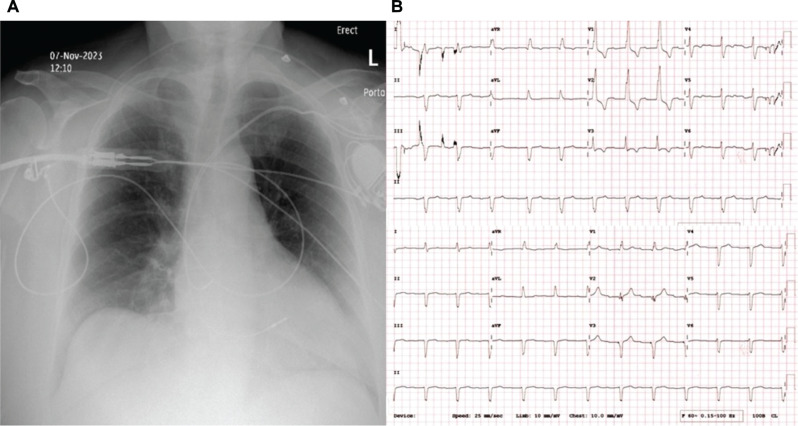
Right ventricular lead position and QRS axis shift in the second patient. **A:** Chest X-ray anteroposterior view with right ventricular lead position. **B:** Electrocardiograms before (top) and after (bottom) pacing, showing a QRS axis shift in leads I and aVL.

Post-pacing QRS morphology varied. A similar morphology to the intrinsic QRS was seen in seven patients, LBBB in four patients, and RBBB in one patient. In two cases, the post-pacing ECG had a negative deflection in leads I and aVL, suggestive of activation of the left side of the septum. The narrowest paced QRS complex corresponded to identification of a discrete spike potential in all 12 patients. **Figures 2** and **3** show ECG and chest X-rays of two patients. Additionally, the optimal lead location was predominantly in the mid-septal (n = 6) and RV outflow tract (n = 6) regions. The mean QRS duration after pacing (121.5 ± 14.9 ms) was not significantly different from the pre-pacing mean QRS duration (118.2 ± 23.5 ms) (*P* = .265; 95% confidence interval, −16.54 ms; mean difference, −5.75 ms). The mean fluoroscopy time for the 12 patients was 3.16 ± 0.56 min, and the mean procedure time was 42 ± 22 min. These times were not significantly different from our standard fluoroscopy and procedure times. All times given here are for a single operator. No postoperative complications were encountered in any patient of this series. At routine short-term follow-up, there were also no complications recorded.

## Discussion

This report of a small early series demonstrates that a narrow paced QRS can be achieved by detailed mapping of the RV endocardium, seeking a spike potential close to the QRS at the onset of the current of injury. The septal outflow tract and mid-septal regions were the best sites. The technique uses standard pacing leads without delivery systems. This pacing method reduces procedure times compared to LBBA and His pacing and is similar to a standard dual-chamber pacemaker implant. If these findings can be widely reproduced, physiological pacing of the ventricles will be delivered relatively easily, safely, and inexpensively.

This technique identifies a location in the septum where there is a spike potential at the onset or shortly after the onset of the current of injury. At this site, the paced QRS is similar in duration to the pre-pacing QRS with a varied morphology. This, however, is not achieved in all patients, with the best location varying from patient to patient. Mapping the RV septum at various locations is crucial for identifying the site that yields the spike potential and the narrowest paced QRS complex. The potential that is seen as a part of the current of injury may reflect subendocardial Purkinje fibers, which are not always present. Of note, there were sites with a spike potential that were not associated with a narrow paced QRS. The outcome achieved may depend on the individual distribution of the conduction system. In the four patients in whom a narrow QRS could not be achieved, it was difficult to identify specific characteristics to determine the reason for this outcome. This method may be applied to a broad population of patients, including those without significant intrinsic conduction system abnormalities, when ventricular pacing is anticipated as a backup, eg, sick sinus syndrome. However, the practicality of this technique will be in the hands of others, and its effects on ventricular function will require a detailed study.

Despite careful mapping in the RV, it is not yet clear whether the left bundle is engaged by this technique. However, without the use of a sheath, it is unlikely that the electrode will penetrate the septum by more than ~2 mm.

This series of 16 consecutive patients used ECG mapping of the RV septal endocardium to locate a spike potential most often at the onset of the current of injury followed by pace mapping to show the narrowest QRS. The varied QRS morphology may reflect the state of the existing conduction system and the connection of fibers engaged with the rest of the intrinsic conduction system. A negative QRS in leads 1 and aVL in two patients may reflect these connections, resulting in early activation of the left side of the septum. A narrower QRS with higher stimulation outputs can also suggest this relationship in some patients. In patients with pre-existing RBBB, pacing in the RV outflow tract septum may have further benefit. This was not evaluated in the study but merits further evaluation.

The deleterious effects of standard RV pacing are well established. Standard RV pacing creates a significant delay in activation of regions of the left ventricle with significant widening of the QRS and an LBBB pattern of paced QRS.^1,2,9^ In addition to changes in ventricular activation, there is associated significant deterioration of systolic function.^1–3,10^ Initially, cardiac resynchronization therapy was employed to reduce such deterioration by the addition of an LV lead.^11^ His-bundle pacing was later employed to further minimize delayed LV activation. More recently, LBBA pacing is widely used to access the LV conduction system, having been found technically easier than the His-bundle pacing and, also, associated with lower stimulation thresholds.^12^ However, these alternative sites of pacing need specialized leads and delivery systems with longer procedure times.^13^ There are procedural complications reported with LBBA pacing, as the lead must be at the correct depth and not penetrating the LV cavity.^14^

Comprehensive knowledge of conduction system anatomy and histology is pivotal, especially in the context of pacing techniques such as this one. The His bundle, a unique strand of specialized myocardium facilitating electrical communication between the atria and ventricles, consists of distinct portions. The initial segment is atrial, and then it penetrates the membranous septum, entirely encased by the central fibrous body, occurring either superiorly or inferiorly within the triangle of Koch.^15^ Subsequently, the non-branching portion follows a variable course in the muscular ventricular septum, while the branching portion is closely associated with the aortic ring. There, it gives rise to the left and right bundle branches. The left bundle branch originates inferiorly to the membranous septum, exhibiting a band-like structure with trifascicular branching into septal, anterior, and posterior fascicles.^16^ Conversely, the right bundle branch is a thin cord-like structure taking a short intramuscular course within the septum before emerging in the subendocardium of the RV at the base of the medial papillary muscle.^17^ After the division of the left bundle into the fascicles, these fascicles arborize. These fibers may be the part of the conduction system that is captured, as evidenced by the finding of a narrow QRS. Further study is required to determine what part of the conduction system is being accessed by sophisticated techniques such as frequency analysis of the signal.

### Limitations

This study is observational, without validation of the potentials identified except by the technique employed. It highlights the possibility of achieving a narrow paced QRS from the right side of the septum. It is assumed that the narrowest QRS will be associated with the best possible ventricular function. Hemodynamic measurements were not obtained in any patient at the time of implantation. Long-term hemodynamic data are also needed to validate any advantage of this technique. The study lacks long-term clinical data on thresholds and outcomes with respect to clinical improvement. These data will be available once the patients have reached adequate follow-up durations, which was not in keeping with the objective of the present study. Another limitation is that this study is based on an assumption that the hemodynamic advantage of a narrow paced QRS duration will offset a change in the activation sequence.

## Conclusion and clinical implications

This approach toward pacing at selected septal sites within the RV opens a possibility of physiologic pacing from the right side of the septum. By avoiding the need for specialized leads and sheaths, our method simplifies the implantation procedure, making it more accessible, efficient, and less expensive. The consistent identification of sites by a discrete potential most often at the onset of the current of injury was associated with a surface QRS that has a narrow paced QRS. Engagement of the intrinsic conduction system may not be possible in all patients depending on the distribution of the subendocardial fibers and their connection with the rest of the more proximal conduction system.

We suggest that this technique may be an alternative strategy to left bundle area pacing because of its relative ease and using standard leads without specialized sheaths. This has a potential of being financially viable, especially as the reimbursement diagnosis-related group is that of a standard pacemaker. Given that the standard of care is now left bundle area pacing with the limitation of RV pacing, long-term outcomes of the present technique will have to be compared with those of left bundle area pacing in terms of hemodynamics, quality-of-life measures, and prevention of pacing-induced cardiomyopathy.
